# A case report of a rare genetic mutation (LMNA-C.185G>C, p.Arg62Pro) associated with dilated cardiomyopathy in a Han Chinese child

**DOI:** 10.3389/fcvm.2024.1450246

**Published:** 2024-09-26

**Authors:** Xiaolin Xu, Tianying Chang, Yan Luo, Lisha Wang, Xiaodan Wang, Jiaxin Shi, Aidong Liu, Jiajuan Guo

**Affiliations:** ^1^ Cardiovascular Department, The Affiliated Hospital of Changchun University of Chinese Medicine, Changchun, China; ^2^ College of Chinese Medicine, Changchun University of Chinese Medicine, Changchun, China; ^3^ EBM office, The Affiliated Hospital of Changchun University of Chinese Medicine, Changchun, China; ^4^ Patient Services Department, The Affiliated Hospital of Changchun University of Chinese Medicine, Changchun, China; ^5^ Cardiovascular Department, The Third Affiliated Hospital of Changchun University of Chinese Medicine, Changchun, China

**Keywords:** pediatric, heart failure, dilated cardiomyopathy, muscular dystrophy, LMNA gene mutation

## Abstract

Dilated cardiomyopathy (DCM) remains an enigmatic myocardial disorder characterized either by enlargement of either the left or right ventricle or both and reduced contractility, posing a significant burden on pediatric populations as a leading cause of cardiac-related mortality and morbidity. This paper presents a compelling case of DCM in a Han Chinese child whose genomic analysis unveiled a novel LMNA-C.185G>C (p.Arg62Pro) variant. Over a meticulous 3-year clinical follow-up, spanning ten outpatient consultations and hospital admissions since the initial diagnosis, the patient exhibited a progressive emergence of various cardiac conduction anomalies closely mirroring LMNA-associated phenotypes. Delving into a comprehensive review of the patient's 14-year medical journey and familial history, antecedent signs of muscular dystrophy (MD) predated DCM onset. Familial scrutiny revealed a lineage marred by muscular atrophy, with the patient's maternal grandmother having a history of muscular dystrophy and an episode of DCM, necessitating cardiac transplantation in the patient's uncle at age 37. This scenario illuminates the intricate interplay between LMNA-associated diseases and genetic predisposition. Timely identification of etiological triggers stands paramount in DCM management. Beyond conventional genetic scrutiny, leveraging novel serum biomarkers such as anti-heart muscle antibodies (AHA) remarkably enhanced diagnostic precision. Notably, personalized therapeutic interventions comprising prednisolone regimens and intravenous immunoglobulin infusions precipitated marked amelioration in heart failure symptoms and serum biomarker profiles. It is noteworthy to identify this novel genetic locus within the Han Chinese populace, underscoring the imperative of expanding the LMNA mutation repository within this demographic cohort. Early recognition of clinical manifestations and etiological cues in pediatric DCM heralds a paradigm shift in risk prognostication and individualized therapeutic interventions, underscoring the profound significance of precision medicine in combating rare familial cardiomyopathies.

## Introduction

Pediatric cardiomyopathy stands as a prevalent contributor to impaired heart function and sudden cardiac death in children. Dilated cardiomyopathy (DCM), a variegated myocardial affliction characterized by the expansion of either the left, right, or both ventricles coupled with diminished myocardial contractility, represents the predominant form of pediatric cardiomyopathy, encompassing 51%–59% of cases, with an annual incidence ranging from 0.18 to 0.73 per 100,000 individuals (1). Males exhibit a higher incidence than females, with a disproportionate risk of sudden death. DCM's precise etiology and pathogenesis remain elusive, compounded by multifaceted pathogenic factors. Treatment primarily revolves around forestalling or managing heart failure; as there are no effective interventions, DCM results in a bleak prognosis. The early causative diagnosis with genetic sequencing technology and AHA clinical examination is of great value for the precise treatment and prevention of cardiomyopathy in children, and it could also predict the risk of sudden death and mortality in DCM.

### Clinical presentation

The proband was a 12-year-old boy. He was admitted to our hospital on October 3, 2021, due to “decreased exercise tolerance and endurance, and cough for one month, aggravated with bilateral lower extremity edema for 17 days”.

Past medical history: The child was noted to have limited dressing activities at 5–6, which was not given much attention. At the age of 10, restricted neck flexion, limited rotation to the left and right, a rigid spine, a backward tilt of the neck and shoulders when standing, elbow contractures, requiring assistance for movement, inability to bend, waddling gait, significant weakness, and inability to dress independently were observed. Except for occasional outings, the child typically remained in a side-lying or prone position at home, with muscle wasting. Five years ago, a diagnosis of scoliosis and atlantoaxial subluxation was made in the local hospital. Since the onset in 2021, the child has been unable to walk independently and predominantly uses a wheelchair for mobility.

Family history: The patient's uncle (father's cousin) underwent a heart transplant due to DCM. He passed away 5 years after the heart transplant at the age of 42. The patient's maternal grandmother has a history of muscular dystrophy and an episode of DCM.

The child began experiencing nocturnal paroxysmal coughing without apparent triggers in August 2021, accompanied by fatigue and abdominal pain after walking approximately 200 m. These symptoms were initially disregarded, and no comprehensive medical assessment was sought. On September 16, 2021, the symptoms worsened following exertion, accompanied by a productive cough with pinkish sputum, nocturnal dyspnea, and bilateral lower limb edema. Within 2 days, the child sought consultations with two tertiary hospitals’ cardiovascular specialists (Changchun, China), where echocardiography revealed cardiomegaly and reduced ejection fraction, with a minimum recorded LVEF of 25.6%, indicative of diffuse left ventricular hypokinesia. Consequently, on September 23, 2021, the child was immediately referred to a cardiovascular specialist hospital (Beijing, China), where echocardiography and cardiac magnetic resonance imaging revealed global cardiac enlargement and positive findings consistent with DCM accompanied by pleural and peritoneal effusions. Admission laboratory investigations, including cardiac enzymes, myocardial injury markers, and BNP levels, all indicated abnormalities (Table 1). Epstein-Barr virus (EBV) and cytomegalovirus nucleic acid quantification tests returned normal results. Electrocardiography (ECG) displayed sinus rhythm with abnormal Q waves and T-wave changes. Coronary artery ultrasound revealed no significant abnormalities in coronary artery position or morphology. Prompt initiation of heart failure-specific treatment was admitted, and the patient improved after treatment.

**Table 1:** Cardiac enzymes and cardiac biomarkers.

Number of hospitalizations	Date	BNP (ng/ml) (reference value)	NT-proBNP (pg/ml) (reference value)	High-sensitivity troponin I (ng/ml) (reference value)	TnT (ng/ml) (reference value)	Myo (ng/ml) (reference value)	CK (U/L) (reference value)	CK-MB (U/L) (reference value)	LDH (U/L) (reference value)	AST (U/L) (reference value)	HBDH (U/L) (reference value)	Anti-cardiolipin antibody (G/A/M)	Coxsackie virus IgM
Third	2021-09-24	841 (0–100)	2,581 (<150)	0.051 (0–0.034)		210.39 (0–140.1)	280 (0–200)	9.90 (0–5)	287 (0–250)	37 (15–40)			
	2021-09-28	427	1,667	0.06	0.052 (<0.014)	123.12	188	7.22	230	37			
Fourth	2021-10-03		2,790 (0–450)		0.043 (<0.01)	344 (0–70)	453 (1–310)	27 (0–24)	270 (120–250)	55 (14–44)	227 (0–182)	Negative	Negative
Fifth	2022-09-29		1,985 (<150)	56.5 (1.5–19)			537 (0–164)	17.6 (<25)	343 (110–290)	38.73 (12–37)	201 (72–182)		Negative
	2022-10-07		1,281				314	19.2	293	40.55	187		
	2022-10-10		3,699				335	17.1	295	36.23	179		
	2022-10-17		2,385				163	24.6	314	27.36	190		Negative
Sixth	2022-12-23		5,432 (0–450)			227.02 (0–48.8)	3,179 (55–170)	30.8 (0–16)	802 (120–246)	101 (12–37)			
Seventh	2022-12-25		4,223										
	2022-12-26					490.40	3,877	118	827	89	642		
	2022-12-28		8,738				2,367	64					
Eighth	2023-01-06		27,474			415.20							
	2023-01-08		>35,000				1,708	42	455	71	366		
	2023-01-16		4,060										
Ninth	2023-02-22		3,457			294.80	1,128						
	2023-02-27		2,210										
	2023-03-05		2,117										
Tenth	2023-08-23		23,444			490.5	1,432	55	447	26	352	490.5	

The third admission was at Fuwai Hospital Chinese Academy of Medical Sciences (Beijing, China). The fourth, seventh, eighth, ninth, and tenth admissions were at the Affiliated Hospital to Changchun University of Chinese Medicine (Changchun, China). The fifth admission was at the National Children's Medical Center Children's Hospital of Fudan University (Shanghai, China). The sixth admission was at Changchun Children's Hospital (Changchun, China).

After discharge, the child's edema subsided, but there was no improvement in exercise tolerance or coughing. The child's parents sought traditional Chinese medicine treatment, and on October 3, 2021, they visited the Affiliated Hospital of Changchun University of Chinese Medicine (Changchun, China). Upon admission, serum anti-myocardial phospholipid antibodies were within normal limits, while Coxsackie virus IgG was positive. Cardiac enzyme levels were elevated (glutamic transaminase 55 IU/L, lactate dehydrogenase 270 U/L, creatine kinase 453 IU/L, creatinase isoenzyme 27I U/L, hydroxybutyrate dehydrogenase 227 IU/L), as were troponin T (TnT: 0.043 U/L) and BNP levels (Table 1). ECG showed sinus rhythm with the left anterior fascicular block. Detailed inquiry revealed no history of upper respiratory or gastrointestinal virus infections in the 1–3 weeks preceding the onset of symptoms. The child received symptomatic treatment for heart failure. The patient's condition remained stable until September 2022.

In September 2022, the child experienced recurrent generalized edema, pleural effusion, dyspnea, palpitations, and oliguria throughout the illness. The family sought medical attention again at the National Children's Medical Center Children's Hospital of Fudan University (Shanghai, China) to clarify the cause of heart failure. Tests for ANA-ENA series autoantibodies, Coxsackie virus IgM, and a respiratory pathogen panel showed no abnormalities. Tests for myocardial antibodies, including β1-receptor antibodies, anti-calcium channel antibodies, and anti-myosin heavy chain antibodies, were all positive. ECG findings suggested ectopic rhythm, atrial fibrillation, and ventricular escape beats. The peripheral blood macro-genome was recommended to be investigated further to identify the infectious pathogen, but the child's parents refused. Family whole-exome sequencing (WES) revealed a novel missense mutation in the LMNA gene (NM_170707.4: c.185G>C, p.Arg62Pro). Throughout the illness, treatment with anti-heart failure medications combined with glucocorticosteroid and intravenous immunoglobulin. After the course, symptoms and echocardiography showed improved cardiac function.

After discharge from the National Children's Medical Center Children's Hospital of Fudan University (Shanghai, China), the child discontinued glucocorticosteroid treatment on their own, and 3 months later, he had a recurrence of heart failure. On December 23, 2022, the child sought treatment at a local pediatric tertiary hospital (Changchun, China) and received symptomatic treatment. On 25 December 2022, the patient re-visited our hospital, was given treatment to correct his heart failure, and was discharged on 29 December with improvement.

Following this discharge, the patient experienced decreased urine output. On January 5, 2023, he developed anuria, dyspnea, and an inability to lie flat. The patient underwent hemodialysis treatment in the nephrology department due to acute kidney failure, hyperkalemia, and cardiogenic shock. The patient had multiple episodes of acute heart failure during this course.

On February 22, 2023, after a pneumonia infection, the patient manifested recurrent generalized edema, dyspnea, and orthopnea. They sought medical attention again at our hospital's cardiology department. Throughout the illness course, he underwent abdominal paracentesis drainage procedures and symptomatic treatment, leading to symptom improvement. The patient had his last outpatient visit on 23 August 2023 at our hospital (NT-proBNP 23,444 pg/ml, Myo 490.50 ng/ml, CK 1,432 U/L, CK-MB 55 U/L, hydroxybutyrate dehydrogenase(HBDL) 352 U/L, lactate dehydrogenase (LDH) 447 U/L, AST 26 U/L) (Table 1). The patient's mother subsequently declined our telephone follow-up visit.

### Laboratory tests

**Figure 1:**
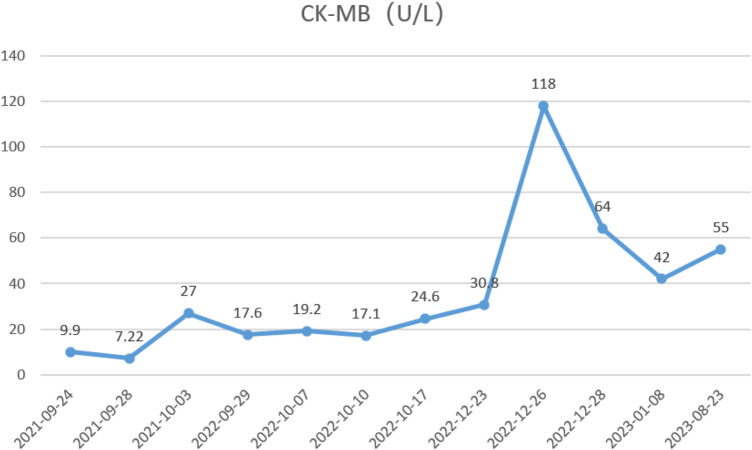
The patient's NT-proBNP level from 2021/9/24 to 2023/8/23.

**Figure 2:**
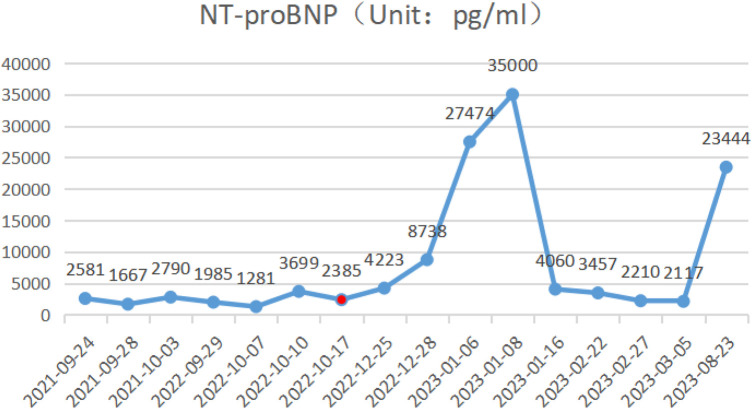
The patient's CK level from 2021/9/24 to 2023/8/23.

**Figure 3:**
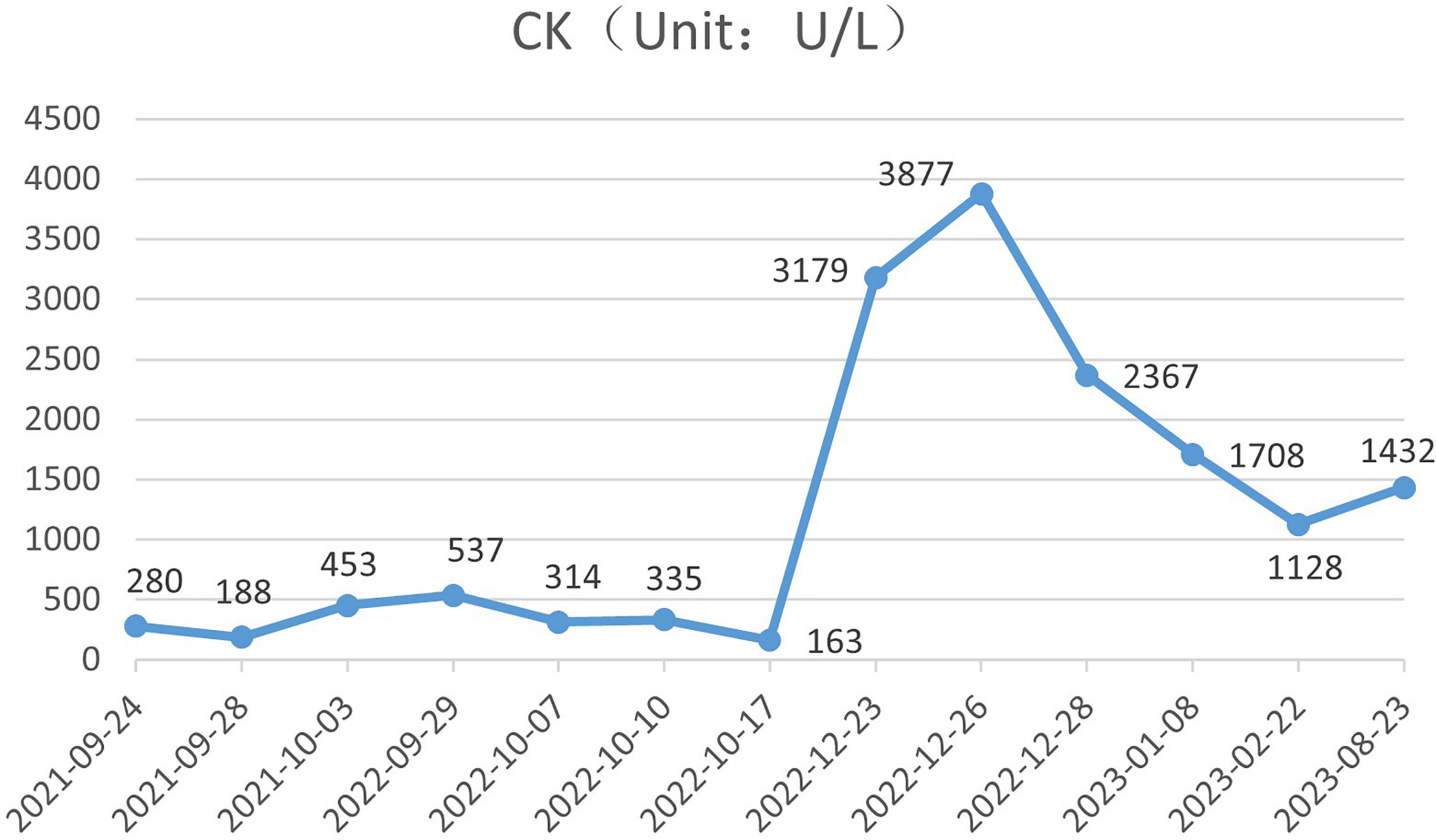
The patient's CK-MB level from 2021/9/24 to 2023/8/23.

During the child's ten visits for medical consultation following the fifth hospitalization (in September 2022, National Children's Medical Center Children's Hospital of Fudan University, Shanghai, China) and treatment of prednisone and intravenous immunoglobulin, CK (Figure 1) and CK-MB levels (Figure 2) approached normal values, and symptoms of heart failure improved. However, CK levels remained relatively high. The patient's NT-proBNP level from 2021/9/24 to 2023/8/23 was shown in Figure 3.

### Metabolic screening

On the fifth visit to the hospital on September 29, 2022, 2-oxoglutaric acid detected more organic Acids in Urine, aconitate acid significantly increased, and citric acid increased. Blood amino acid and carnitine results showed normal.

On the ninth visit to the hospital on January 9, 2023, blood ammonia was 85 umol/L.

### Electrocardiogram

**Figure 4:**
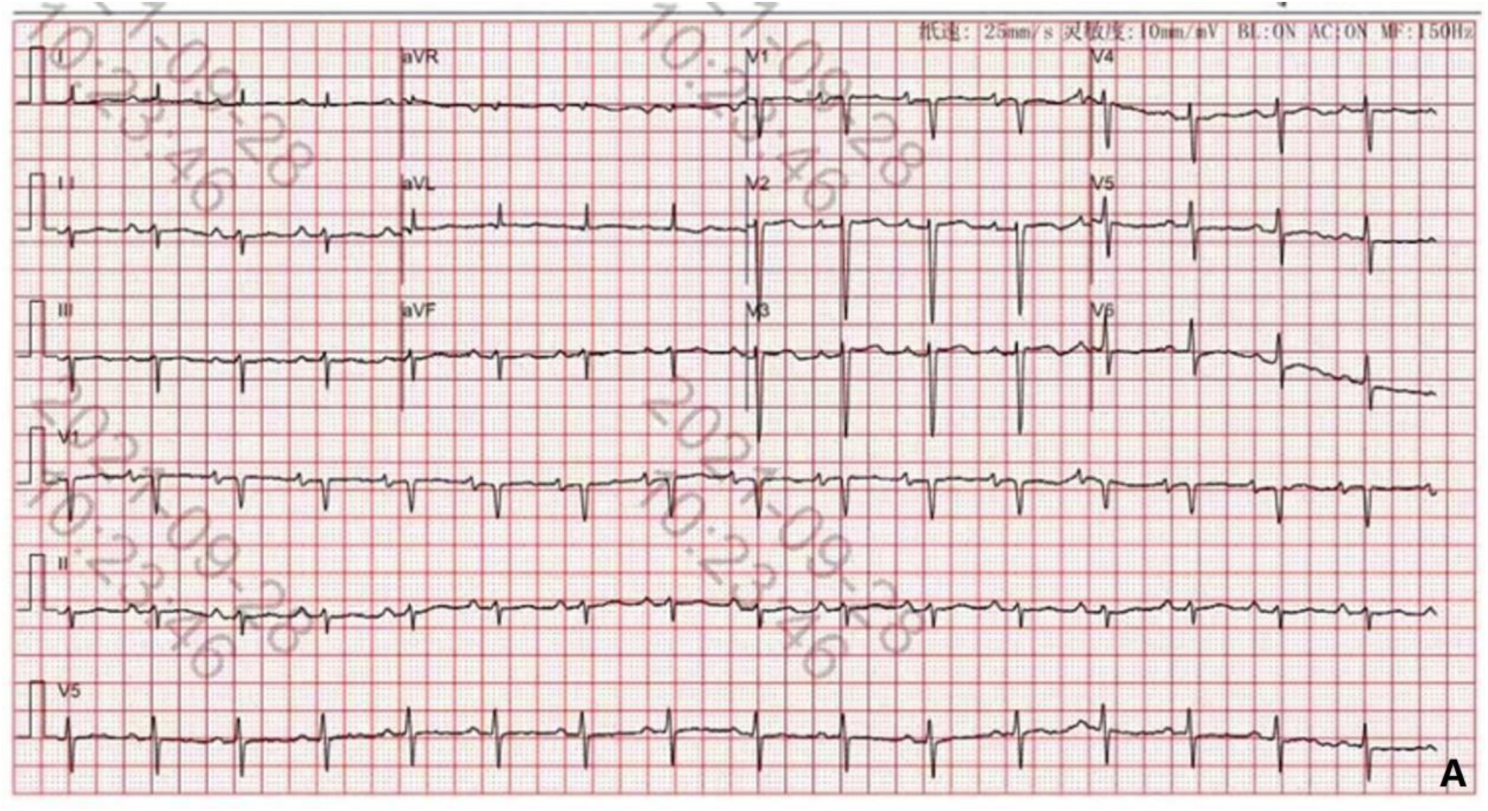
ECG on September 28, 2021.

The child's first hospitalization ECG, taken on September 28, 2021, showed sinus rhythm, left anterior fascicular block, and T-wave changes (Figure 4).

**Figure 5:**
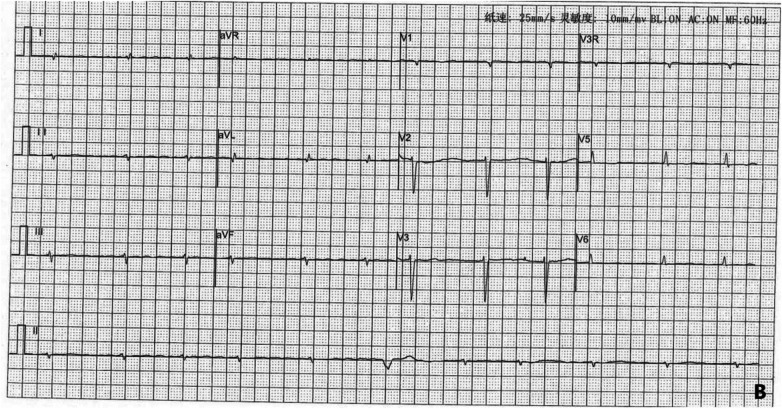
ECG on September 29, 2022.

On September 29, 2022, during the child's fifth hospitalization, the ECG revealed ectopic rhythm, atrial fibrillation, and ventricular escape beats (Figure 5). The patient's ECG changes from 2021/9/23 to 2023/2/22 was shown in Table 2.

**Table 2:** ECG results from September 23, 2021, to February 22, 2023.

Number of hospitalizations	Date	Rhythm	QRS		ST-T	Voltage	Diagnosis
Third	2021-09-23	Sinus rhythm	Abnormal Q wave		T-wave changes		
	2021-09-26	Sinus rhythm					Atrial premature beats, occasionally seen in couplets, occasionally seen in triplets, occasionally seen in pairs; paroxysmal atrial tachycardia; occasional premature ventricular contractions (PVCs); atrial escape beats can be seen
Fourth	2021-10-03	Sinus rhythm	Poor R-wave progression in the chest leads		T-wave changes		Sinus tachycardia; left anterior fascicular block
	2021-10-11	Sinus rhythm	Poor progression of R waves in the chest leads		ST-T changes		Isolated premature ventricular contractions (PVCs); isolated premature atrial contractions (PACs)
Fifth	2022-09-27	Junctional escape rhythm	I in lead aVL shows qR pattern and leads V1 and V3R show QS patterns		Inverted I and aVL, low voltage in V5 and V6	Low voltage	
	2022-09-29	Ectopic rhythm	Poor R-wave progression in the anterior precordial leads; qR pattern in I and aVL, QS pattern in V1 and V3R			Low voltage	Atrial fibrillation,ventricular escape beat
	2022-10-08	Ectopic rhythm	Poor R-wave progression in the anterior wall, qR pattern in leads I and aVL, QS pattern in leads V1 and V3R.			Low voltage	Atrial fibrillation
Seventh	2022-12-25	Ectopic rhythm	Poor R-wave progression in the chest leads.	Abnormal Q waves.	ST-T changes		Atrial fibrillation
Ninth	2023-02-22	Ectopic rhythm	Abnormal Q waves.	Abnormal Q waves.	ST-T changes		Atrial fibrillation

### Echocardiography

**Figure 6:**
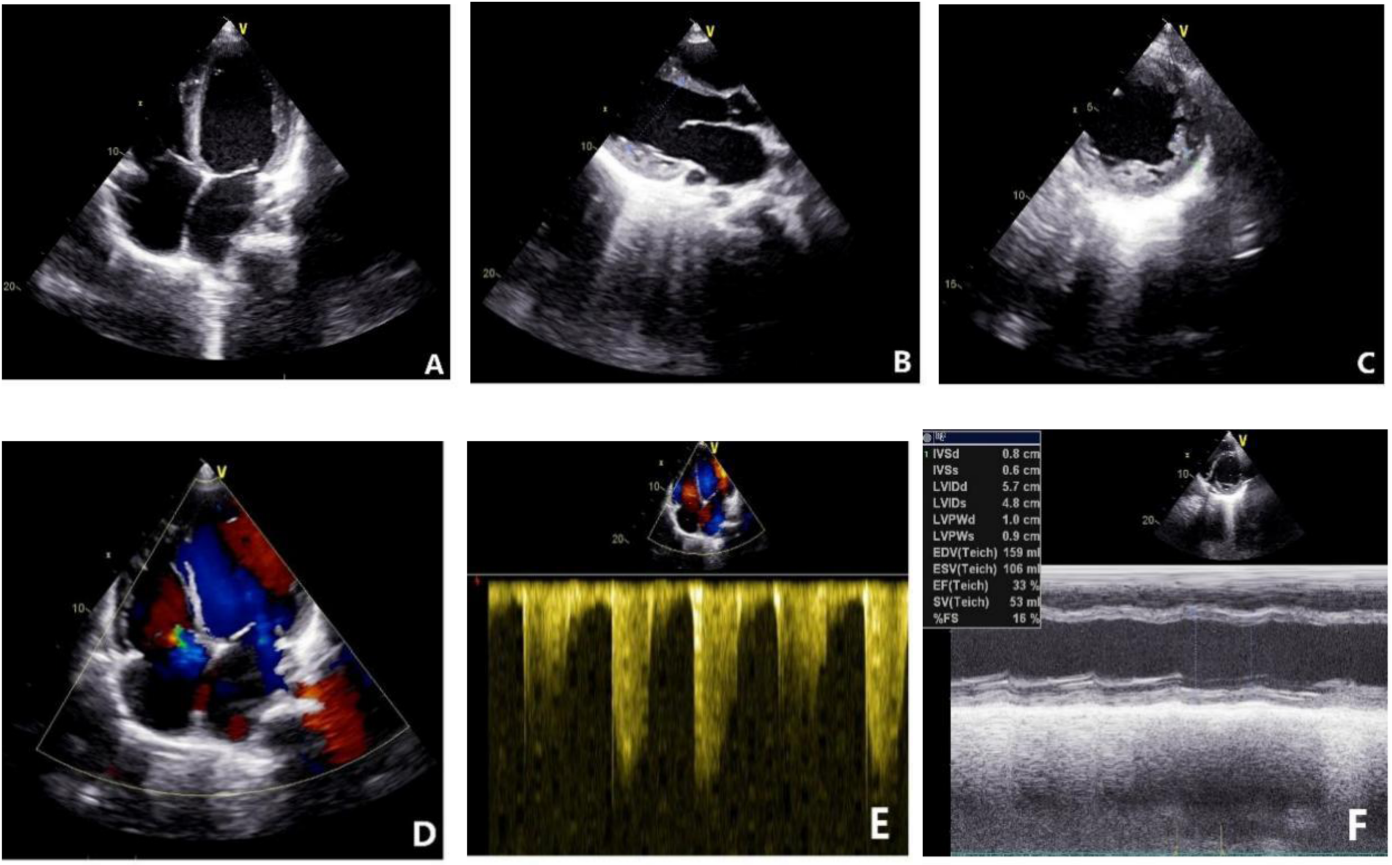
Echocardiogram results on October 14, 2021. **(A)** Four-chamber view showing global enlargement of the heart. **(B)** Long-axis view of the left ventricle indicating a left ventricular end-diastolic diameter of 51.60 mm. **(C)** Increased number of trabeculations in the mid to lower segments of the left ventricular lateral wall, with a trabecular height of 8.5 mm at the lateral wall. **(D)** Moderate regurgitation of both the mitral and tricuspid valves. **(E)** Indirect estimation of pulmonary artery systolic pressure of 40 mmHg. **(F)** Reduced cardiac function with an ejection fraction (EF) of 33%.

**Figure 7:**
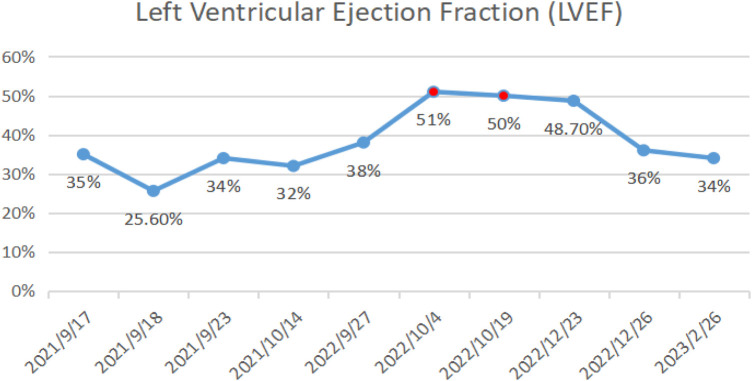
LVEF results from September 17, 2021, to February 22, 2023.

On October 14, 2021, echocardiography results showed an enlarged heart, reduced cardiac ejection fraction, and myocardial fibrosis (Figure 6, calculated with the M-mode ultrasound method and modified Simpson method). We recorded the change in LVEF from 17 September 2021 to 22 February 2023 (Figure 7).

### Coronary vascular ultrasound

The coronary artery ultrasound examination on September 23, 2021, showed that the origins of the left and right coronary arteries are in their normal positions. The right coronary artery arises from the right coronary sinus. In contrast, the left coronary artery arises from the left coronary sinus, branching into the left anterior descending artery and circumflex artery. The luminal diameters at the proximal ends are within normal range. Color Doppler flow imaging (CDFI) shows no significant abnormal blood flow signals in the proximal portions of the left and right coronary arteries. There are no obvious abnormalities in the positions or courses of the left and right coronary arteries.

### Chest x-ray examination

**Figure 8:**
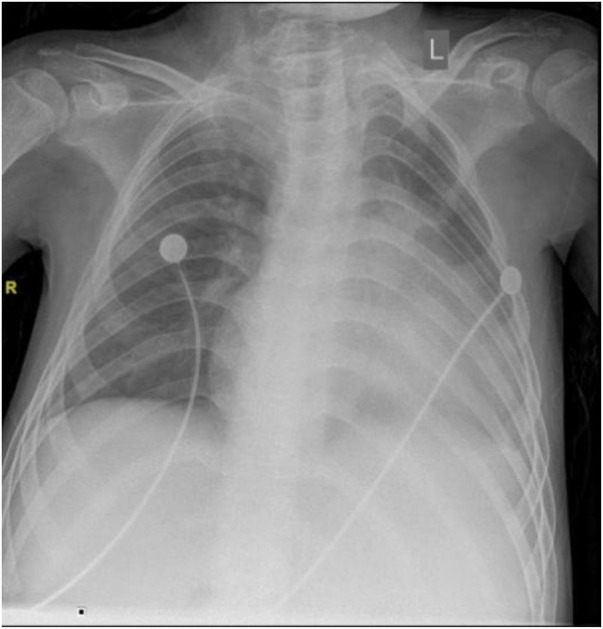
Chest x-ray on September 23, 2021.

The chest x-ray examination on September 23, 2021, showed a slight increase in coarse and disordered pulmonary markings bilaterally, enlarged heart, straight cervical spine curvature, and scoliosis of the spine (Figure 8).

### Cardiac enhanced MRI

**Figure 9:**
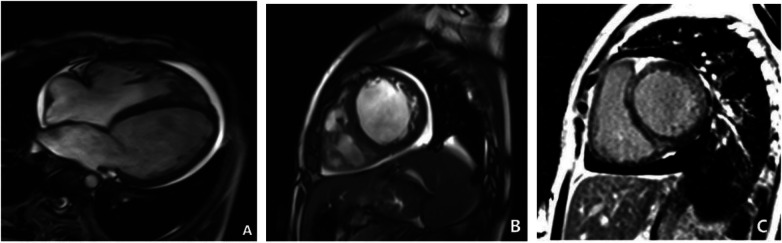
Cardiac MRI on September 23, 2021. **(A**,**B)** Show short-axis and four-chamber cine images that suggest left ventricular enlargement, reduced wall motion, and increased free wall thickness. **(C)** Showed delayed enhancement imaging in the short-axis view, which revealed linear enhancement between the mid-septal myocardium and the mid-lateral left ventricular wall.

On September 27, 2021, the patient underwent a cardiac-enhanced MRI to assess myocardial tissue characteristics (Figure 9). The report indicates significant enlargement of the left atrium and left ventricle. The walls of various segments of the left ventricle are at the normal lower limit or slightly thinned, with significantly reduced contractile motion and mild hypertrophy of the lateral wall's distal trabeculae. The left ventricular outflow tract is unobstructed. The right atrium and right ventricle show mild enlargement with minimal tricuspid regurgitation. No abnormal fat infiltration is observed in the right ventricular wall, but there is reduced contractile motion with an unobstructed right ventricular outflow tract. The aortic valve function is normal. There is a small amount of fluid signal in the pericardial cavity. Myocardial first-pass perfusion: no obvious perfusion reduction or defects; delayed enhancement scan: linear enhancement seen between the interventricular septum's near-mid segment and the left ventricular side wall's near-mid segment, indicating local myocardial fibrosis. The MRI showed that global cardiac enlargement, predominantly affecting the left heart, with reduced biventricular systolic function and localized myocardial fibrosis in the left ventricle, suggestive of non-ischemic cardiomyopathy, with DCM being a significant possibility, and a small amount of pericardial effusion.

### Genetic analysis results

The patient's mother had six pregnancies, with two successful pregnancies. The child was born to his mother in the 2nd birth of her 6th pregnancy, prematurely, by cesarean section, and weighed 4 kg at birth. The mother did not have any infections or use medication for fever during the first 3 months of pregnancy. The patient's sibling, a sister with the same parents, is healthy and denies any heart disease; all examinations showed normal cardiac function and rhythm. In the patient's family, their uncle (cousin of the father) underwent heart transplantation due to DCM on March 17, 2010, and died 5 years post-transplantation at the age of 42; however, genetic testing results are unknown. A novel missense mutation (NM_170707.4: c.185G>C, p.Arg62Pro) in the LMNA gene was detected through whole exome sequencing in the proband. Data were collected from the patient and third-degree relatives; the proband's parents have normal phenotypes, and both entire exome sequencing and Sanger sequencing were negative for this variant. The proband's sister did not undergo genetic testing. This mutation c.185G>C, p.Arg62Pro identified in the proband's sample is novel and not recorded in databases like HGCM and gnomAD, with no available literature reports. According to ACMG guidelines, this variant is classified as a likely pathogenic mutation and may be a deleterious genetic mutation. LMNA gene mutations can cause DCM, muscular dystrophy, Mandibuloacral dysplasia, and Hutchinson-Gilford progeria syndrome. Besides cardiac involvement, the patient also presents with extracardiac manifestations of muscular dystrophy. Refinement of electromyography, muscle MRI, and muscle biopsy examinations are recommended, but the family declined. In the patient's family, the grandmother was diagnosed with muscle atrophy in 2010, with electromyography indicating myogenic damage; she was bedridden by 2023, while no signs of muscular dystrophy were present in other family members. Although the pathogenic mutation was detected in the patient, it is absent in the parents’ somatic cells. However, given more than 2 cases of DCM in the family, including the proband, the patient is considered to have familial dilated cardiomyopathy.

### Family chart analysis

**Figure 10:**
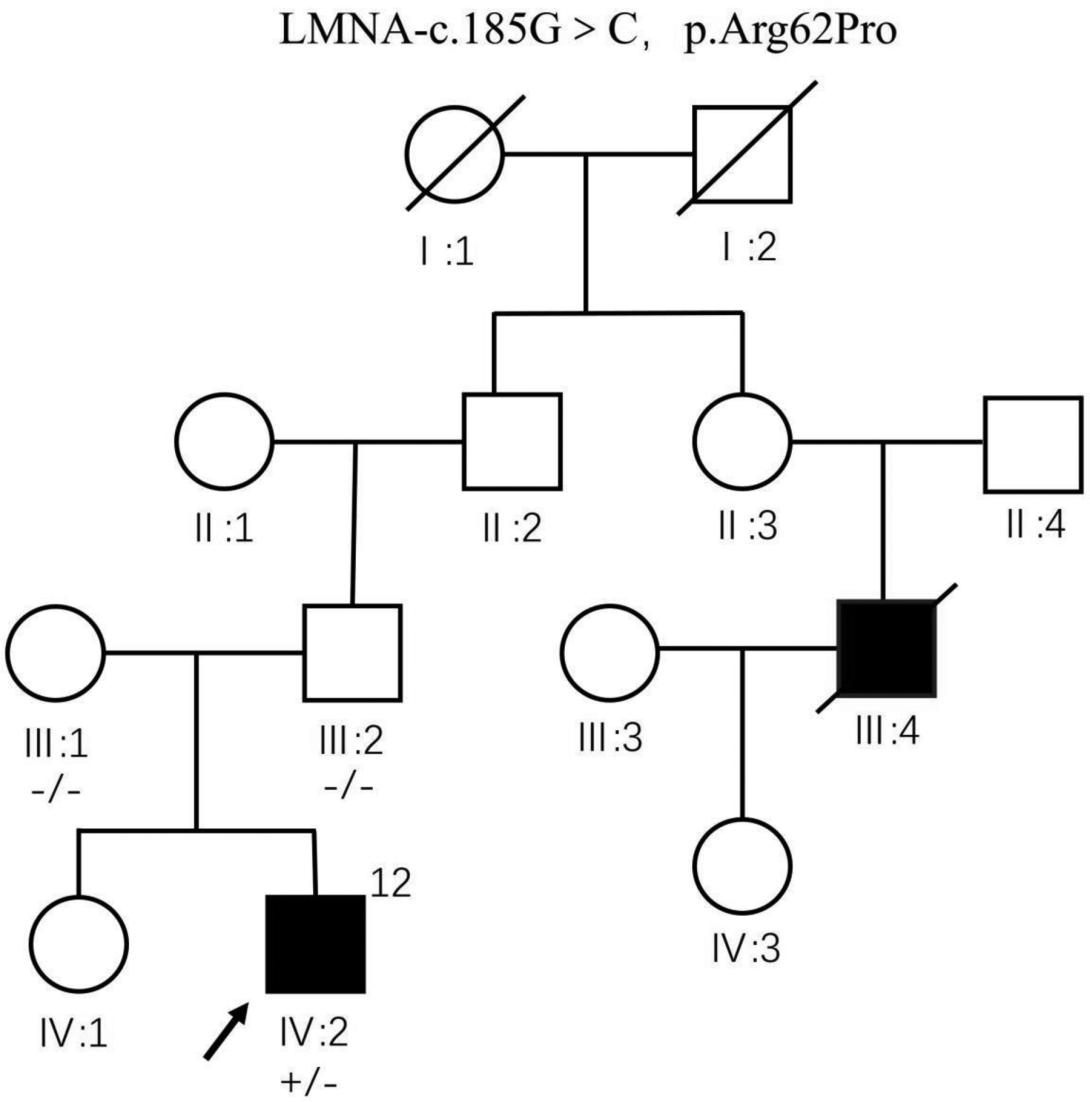
LMNA gene mutation DCM disease family chart.

The predeceased (IV: 2) carries heterozygous variant c.185G>C, p.Arg62Pro of LMNA gene, and no abnormality was seen at the locus of genetic testing performed by father (III: 2) and mother (III: 1). The first uncle of the pre-witness (III: 4) underwent heart transplantation at the age of 37 years for dilated heart disease without genetic testing and died at the age of 42 years. The remaining family members were not genetically tested (Figure 10).

## Discussion

DCM, as a primary type of pediatric cardiomyopathy, can occur during embryonic development or at any age during childhood, differing from adult-onset DCM. In 2023, the American Heart Association published a scientific statement on pediatric cardiomyopathy in the Circulation journal, emphasizing the increasingly evident differences between pediatric and adult cardiomyopathy in etiology, risk factors, pathophysiology, and disease progression (2). The patient has improved clinical symptoms by applying treatment strategies referenced from adult guidelines to pediatric DCM and heart failure. The use of ACE inhibitors and beta-blockers has not significantly improved the survival rates of non-heart transplant pediatric patients (3, 4). Therefore, aside from symptomatic treatment, there is a particular emphasis on etiology-specific treatment for children to prevent and alleviate their cardiomyopathy.

The etiology of pediatric DCM is difficult to determine, with this patient undergoing visits to multiple hospitals to complete relevant examinations. Ultimately, the DCM diagnosis is associated with factors including genetic mutations and autoimmune inflammation. In this case, whole-exome sequencing identified a novel missense mutation in the LMNA gene (C.185G>C, p.Arg62Pro), which was reported clinically for the first time as being associated with DCM. Clinical research has identified at least 60 genes related to DCM, with 12 strong mutation genes among the 19 common mutation genes in DCM. These genes include TTN, LMNA, MYH7, BAG3, TNNT2, FLNC, RBM20, SCN5A, PLN, TNNC1, TNN13, and TPM1 (5). Among them, LMNA is DCM's second major causative gene. Our research suggests that the main gene mutation type in the Han Chinese population is the TTN gene, while LMNA mutations are relatively rare among Asian populations (6).

Lamin A/C-related diseases (LMNA) are a group of human genetic disorders caused by abnormalities in the LMNA gene and its encoded protein, lamin A/C. LMNA mutations are inherited in an autosomal dominant manner. The nuclear lamina, located at the inner layer of the nuclear membrane, is a critical structure for maintaining genomic and cellular mechanical stability and is involved in various biological processes within the nucleus (7). When the LMNA gene mutates, it alters the cellular nuclear structure, disrupting the components of the nuclear lamina and leading to changes in the gene expression profile. This affects various cells and tissues such as bone, muscle, and fat, resulting in conditions including muscular dystrophy, familial partial lipodystrophy, Hutchinson-Gilford progeria syndrome, DCM, and conduction abnormalities. Although these diseases share the same genetic basis, their clinical phenotypes vary significantly, with some overlapping features.

LMNA gene mutation-related DCM is a highly pathogenic and age-dependent disease characterized by a high incidence of atrioventricular conduction block, ventricular arrhythmias, and sudden cardiac death, with rapid disease progression. In this patient's case, the LMNA gene is located on chromosome 1, Chr1 (GRCh37):g.156084894G>C. Over 3 years of follow-up, the clinical manifestations and diagnostic and therapeutic processes of this patient's spontaneous disease onset over 3 years were tracked and summarized. Observation of the disease progression associated with the LMNA gene mutation in this patient showed changes in the electrocardiogram over the 3-year course, evolving from sinus rhythm initially to various premature contractions, conduction blocks, escape rhythms, and atrial fibrillation, accompanied by ST-T changes, low voltage, poor R-wave progression, abnormal Q waves, consistent with the pathogenic characteristics of LMNA mutation, and indicating a significant risk of sudden cardiac death. In this case, only the child and parents had improved genetic testing. Genetic testing was not completed for the remaining family members due to the local hospital's unavailability of genetic testing services. We can only observe the changes in the disease from the clinical findings. We cannot further understand the genetic characteristics of the child's family through genetic testing.

Besides severe cardiac involvement caused by LMNA gene mutation, reviewing the entire course of the patient's illness, in this case, the child developed the disease before school age, with mild symptoms of muscular dystrophy at the age of 5, worsening at ten, and exhibiting heart failure, ventricular enlargement, and arrhythmia at 12. Early signs of muscular dystrophy were present but not adequately recognized. LMNA gene-related muscular dystrophies include LMNA-related congenital muscular dystrophy (L-CMD), autosomal dominant Emery-Dreifuss muscular dystrophy (EDMD2), autosomal recessive Emery-Dreifuss muscular dystrophy (EDMD3), and limb-girdle muscular dystrophy (LGMD) type 1B (8). Based on the onset time, disease characteristics, and extent of cardiac involvement, LMNA gene mutation-induced EDMD had mild skeletal muscle involvement but more severe cardiac involvement. Cardiac conduction system involvement often accompanies DCM and/or heart failure. Throughout the illness, creatine kinase levels were significantly elevated, remaining high even when myoglobin and BNP levels decreased, indicating muscle damage beyond the myocardium, consistent with clinical features of EDMD. Unfortunately, the family refused further orthopedic and muscular examinations during the illness, preventing clarification of whether the patient's muscle damage had other causes. Additionally, the severity of cardiac involvement did not parallel the severity of muscle atrophy and weakness, indicating a progressive disease trend. Literature reports suggest that LMNA-related cardiomyopathies exhibit skeletal muscle phenotypes approximately 10 years earlier (8, 9). In recent years, patients with combined neurological, muscular, and cardiac involvement have constituted a unique group, with those exhibiting both cardiac and neuromuscular signs more frequently experiencing bradyarrhythmias and atrial fibrillation. Cardiac involvement is a common extramuscular manifestation of many hereditary neuromuscular diseases. In this case, the patient exhibited spinal deformity, gait abnormalities, and muscle contractures preceding the onset of DCM, providing clearer observations of the natural progression of LMNA gene mutation and enriching the association between LMNA disease phenotypes and causative genes.

The pathogenic mechanism of DCM caused by LMNA mutations has not been fully elucidated. Therefore, there are currently no specific treatment protocols for LMNA gene-related DCM. Treatment mainly follows the principles of DCM management, aiming to prevent myocardial damage mediated by the underlying pathogenesis, effectively control heart failure and arrhythmias, prevent sudden death, and improve patients’ quality of life and survival rate. Due to the unique characteristics of the pediatric population, conducting clinical trials of drugs poses significant obstacles. Current guidelines for pediatric DCM and heart failure are mainly based on expert consensus, with clinical medication experiences borrowed from adults and low levels of evidence (7). The latest drug treatment directions for LMNA mutation-induced DCM mainly focus on targeted drug research of relevant pathways. For instance, modulation of the AKT/mTOR pathway (sirolimus) (10), MEK1/2 small molecule allosteric inhibitors (PD98059 and selumetinib) (11), and P38a small molecule inhibitors (ARRY-371797) have been shown to improve left ventricular size, function, and fibrosis (12). Professor Joseph Wu's team found that the LMNA K117fs mutation leads to significant calcium homeostasis imbalance at the single-cell level in iPSC-CMs, and abnormal activation of PDGF signaling closely matches the severe arrhythmogenic clinical phenotype observed in LMNA mutation-induced DCM patients. Inhibiting PDGF receptor-β (PDGFRB) activity offers a new drug target and treatment strategy for LMNA mutation-induced DCM (13).

Currently, there is no disease-modifying therapy specifically targeting Emery-Dreifuss muscular dystrophy (EDMD). Treatment primarily involves clinical monitoring and symptomatic management, with no definitive cure. For patients presenting with early clinical phenotypes of muscle wasting, cardiac involvement significantly impacts their quality of life and survival. It is recommended to conduct comprehensive muscle assessments, undergo early genetic testing for disease diagnosis and classification, and regularly assess cardiac disease risk, including electrocardiograms and 24-h ambulatory electrocardiography. Children with LMNA gene mutations have a significantly higher risk of adverse cardiac outcomes (including heart transplantation, death, etc.) compared to those with other mutation types. These children often experience complex arrhythmias and positive myocardial antibodies, leading to end-stage heart failure with a 5-year survival rate of only 50%. The risk of sudden death is extremely high, with a poor prognosis overall.

Guidelines recommend ICD therapy for patients with DCM who have symptoms of heart failure despite >3 months of optimized drug therapy (LVEF <35% and an expected survival of >1 year） (Class I recommendation, Level B evidence) (14). International cardiovascular disease treatment guidelines recommend that patients with LMNA gene mutations undergo ICD implantation to prevent adverse cardiovascular events (15). For children with LMNA mutations, early intervention with pacemakers or cardiac defibrillators is necessary if cardiac conduction abnormalities occur. The latest scientific statement from the American Heart Association (AHA) in June 2023 regarding treatment strategies for pediatric cardiomyopathy states that patients classified as having Stage D heart failure, meaning refractory heart failure despite maximal medical therapy, may benefit from specialized interventions such as mechanical circulatory support, continuous intravenous infusion of positive inotropic agents, heart transplantation, and palliative care or hospice care interventions (2). However, heart transplantation and device-assisted therapies still have limitations in this specific pediatric population.

In this case, while confirming the LMNA gene mutation as the cause, and despite standardized heart failure treatment based on heart failure guidelines, if the child's condition continues to deteriorate, besides enhancing genetic screening for hereditary causes, a comprehensive reevaluation of the underlying causes of DCM in the child should be conducted. For non-ischemic heart failure patients with cardiac enlargement, early detection of AHA-recommended immunodiagnosis for DCM is advised. The child's myocardial antibodies tested positive for anti-β1AR antibodies and anti-L-CaC antibodies, both of which are independent predictors of death and sudden death in DCM patients (5, 16–18). Therefore, for early-stage DCM patients with positive anti-β1AR antibodies and/or anti-L-CaC antibodies, the recommendation is to use beta-blockers and diltiazem therapy to counteract the pathogenic effects of the antibodies. Contraindications for the use of these two drugs include hypotension, bradycardia, and atrioventricular conduction block, with diltiazem being contraindicated in patients with LVEDd ≥7.0 cm and NYHA class II–IV heart failure. Since the child experienced hypotension and heart failure during the disease, diltiazem was not administered. Additionally, for DCM patients testing positive for AHA, combination therapy with immunoadsorption and immunoglobulin supplementation (IA/IgG) is recommended to reduce myocardial damage and delay disease progression.

During the ten visits of this patient, the persistent elevation of cardiac troponin and myocardial enzymes stands out as another characteristic distinguishing this case from other DCM patients. Throughout the illness, comprehensive assessments revealed normal coronary arteries, non-ischemic etiology on cardiac magnetic resonance imaging, features suggestive of DCM on echocardiography, and symptoms of heart failure. Additionally, elevated erythrocyte sedimentation rate and high-sensitivity C-reactive protein were present, viral infection was ruled out, and there were no risk factors such as hypertension, diabetes, hyperlipidemia, or smoking. Persistent elevation of cardiac troponin was observed despite standard heart failure treatment, prompting consideration of inflammatory or immune-mediated inflammatory cardiomyopathy (infl-CMP). Endomyocardial biopsy is the gold standard for diagnosing infl-CMP, and consensus recommends therapy with prednisone and immunosuppression to rebalance inflammatory and anti-inflammatory factors (19, 20), resulting in favorable anti-inflammatory effects and improvement in patient cardiac function. During this patient's fourth episode of illness, administration of prednisolone tablets and intravenous immunoglobulin led to clinical improvement. Cardiac troponin and myocardial enzymes approached normal levels, cardiac function and clinical symptoms improved, and the EF value increased to above 50%. Upon discharge, these medications were discontinued, but upon resuming standard heart failure treatment, a decline in cardiac function and a decrease in EF value were observed, further supporting the possibility of inflammatory cardiomyopathy during this disease.

We communicated with the patient's parents as much as possible and suggested that other family members’ genetic testing should be improved. Still, due to the unavailability of genetic testing in the local hospital, the remaining family members did not improve their genetic testing. We can only observe changes in the disease from the clinical phenotype, and we cannot use genetic testing to further understand the genetic characteristics of the child's family, which is the limitation of this case report.

We believe that in future encounters with the same type of patient, early communication with families should be made to achieve as complete a collection of clinical data for genetic testing as possible. The AHA antibody test has demonstrated significant value in identifying and diagnosing DCM. However, the popularity of AHA antibody testing is still insufficient, and timely testing of this indicator is crucial for early disease detection.

From the patient's perspective, the child and his parents experienced multiple hospital visits across the region and continued to deteriorate despite standardised heart failure treatments in all hospitals. During this process, the most urgent wish of the child's family is to identify the cause of the disease in order to find an effective treatment plan to alleviate the child's suffering. With the confirmation of the LMNA gene mutation and the immunoinflammatory etiology of the disease, the family has a clearer understanding of the disease, and the direction of treatment has been clarified. However, there is no specific treatment for LMNA gene mutation cardiomyopathy, and the parents reported a significant improvement in their condition after using Chinese herbal medicine decoction and immunosuppressive therapy. This practice suggests that interventions targeting immune factors may play an active role in treating DCM, providing new perspectives and strategies for disease management.

## Conclusion

Pediatric DCM exhibits significant genetic heterogeneity. Early, pathophysiology-based diagnosis, improved genetic sequencing screening, AHA antibody screening, and monitoring of cardiac troponin levels in non-ischemic DCM patients contribute to early clinical treatment, genetic counseling, and disease prognosis assessment.

By reporting a clinical case of DCM in a Han Chinese child with a rare gene mutation (LMNA-C.185G>C, p.Arg62Pro), this study draws attention to the different clinical phenotypes caused by LMNA gene variations. The natural history of myopathy progressing to cardiac damage and related complications was observed. Further investigation into the mechanism of LMNA gene-related pediatric DCM should be conducted, which may reveal the relationship between LMNA genotype and phenotype. Screening for antibodies to AHA is beneficial for the early diagnosis of DCM. This case provides some clinical experience in finding more effective interventions and precise treatments for DCM.

Written informed consent was obtained from the minor legal guardian for the publication of any potentially identifiable images or data included in this article.

## Data Availability

The original contributions presented in the study are included in the article/Supplementary Material, further inquiries can be directed to the corresponding authors.
